# Deaths in Australia from Work-Related Heat Stress, 2000–2015

**DOI:** 10.3390/ijerph16193601

**Published:** 2019-09-26

**Authors:** Richard Gun

**Affiliations:** Adelaide Exposure Science and Health, Faculty of Health and Medical Sciences, University of Adelaide, Adelaide 5005, Australia; richard.gun@adelaide.edu.au

**Keywords:** hyperthermia, heat stroke, occupation, acclimatisation, self-pacing, cerebral oedema

## Abstract

The infrequency of deaths from work-related heat stress may be due to self-pacing, whereby workers adjust their work rate in response to thermal discomfort. Thirteen cases attributed after coronial investigation to work-related heat stress were studied to evaluate the causal contribution of environmental and personal risk factors. Meteorological records and coronial records were examined to estimate environmental and metabolic heat loads and to identify any personal risk factors likely to have contributed to death. Seven deaths occurred in workers within one week of hiring, demonstrating not only the importance of acclimatisation but also the likelihood of compromised self-pacing in recently-hired workers. Personal risk factors identified included intercurrent illness, cardiovascular disease and obesity. Four deaths occurred following indoor work, where the probable critical risk parameter was low air velocity. Cerebral and pulmonary oedema were reported in some autopsy reports, and uncal herniation was found in one case. Modified work rates and close supervision are essential in recently-hired workers. The risk of death from raised intracranial pressure suggests the need for specific remediation of cerebral oedema in hyperthermic individuals.

## 1. Introduction

Death from occupational heat stress is not common. In the U.S., only 13 such deaths were reported for the period 2012–2013 [1]. In Australia, the National Coronial Information System has recorded only 17 cases between 2000 and 2015, even though most workers in the mining, agriculture and construction sectors work outdoors in the hotter regions of Australia [2]. The infrequency of deaths from this cause may be attributable to self-pacing, which is the natural behavioural response to heat whereby discomfort arising from a combination of metabolic and environmental heat load leads to slowing of the work rate [3,4].

This study was based on reports in which the coroner had found death to be attributable to work-related heat stress. The aim was to confirm (or not confirm) the coroners’ conclusion that death was attributable to work-related heat stress and, in those cases where heat stress was found not to be a sufficient cause, to identify other contributing factors.

## 2. Materials and Methods

Details of deaths from work-related heat stress were obtained from coronial records. Seven deaths were the subject of coronial inquests with reports on the public domain and were obtained from SafeWork Australia. Reports of the other deaths were obtained from State coronial offices. The study was approved by the Human Research Ethics Committee of the University of Adelaide.

For each death, weather observations were obtained for the day when exposure occurred (not always the date of death) from the nearest monitoring station of the Bureau of Meteorology (BOM). The observations include air (shade) temperature, dew point, relative humidity, air velocity, and total daily solar exposure. Information on work performed and personal information relating to the deceased worker were obtained from coronial records.

Records of each case were examined: (i) to confirm (or not confirm) the coronial finding that work-related heat stress was a necessary cause of the death, i.e. whether the death would not have occurred but for that level of heat stress; and (ii) to determine whether the level of heat stress was a sufficient cause of the death.

The main criterion to confirm heat stress as a cause of death was heat stroke, identified by a body core temperature greater than 40.0 °C. Where no body temperature was recorded, the decision on whether, on the balance of probability, heat stress was a contributory cause of death was based on computation of metabolic and environmental heat load, likely fluid deficit and maximum sweat evaporative capacity (Emax).

The decision on whether heat stress was a sufficient cause depends primarily on whether other workers performing the same work in the same environment developed heat-related illness: if not, heat stress is self-evidently not a sufficient cause. Where there were no others performing similar work, the decision was based on information from BOM data and from coronial documents on the presence of known risk factors for heat-related death or illness.

BOM data on air temperature and humidity were used to derive the Heat Index, using the calculator of the US National Weather Service https://www.wpc.ncep.noaa.gov/html/heatindex.shtml.

BOM data do not include mean radiant temperature in direct sunlight. An approximation was obtained by assuming that mean radiant temperature in the absence of cloud cover or shade was double the shade temperature, an assumption based on direct observations made by this author in previous field studies [5]. Radiant Heat Load was then estimated from equations derived experimentally by Hertig [6]. Corroboration of the level of radiant heat was obtained by BOM records, based on satellite data, of total daily solar load in kWh·m^−2^.

Metabolic heat load was estimated from the Table of Metabolic Rate according to activity level from the Heat Stress and Strain document of the American Conference of Governmental Industrial Hygienists (ACGIH).

Heat load and consequent fluid requirement from evaporative sweat loss were estimated from combined radiant and metabolic heat load. Convective heat load was not included because of the relatively small gradient between air and skin temperatures.

An equation by Hertig was also used to estimate maximum sweat evaporative capacity (Emax) [6]. This function is derived from air velocity, partial pressure of water vapour (PPA), and a factor for clothing. PPA was computed from dew point on a website of the U.S. National Weather Service https://www.weather.gov/epz/wxcalc_vaporpressure.

## 3. Results

### 3.1. Study Population

Altogether 13 reports were obtained for the period 2000–2015. Four more deaths occurred from work-related heat stress in one State, but the coroner’s office in that jurisdiction declined the request to supply the documentation. All cases but one (#12) were male. Ages ranged from 19 to 72 years, and six subjects were aged 25 years or less. Ten of the deaths occurred in January and one in February, i.e., at the height of the Australian summer, and two in November.

### 3.2. Deaths in Which Heat Stress Was a Causal Factor

Table 1 shows a summary of heat stress parameters and risk factors, and a summary of each case is given in Appendix B.

Heat stress was a probable contributing factor in 12 of the 13 deaths. In five cases, this conclusion was based on a diagnosis of heat stroke, i.e., body core temperature >40.0 °C. In six cases, the worker had already died when found, and no ante-mortem temperature measurement was recorded: in all six of these deaths, the heat stress parameters and the history justify the coroners’ conclusions that death resulted from heat stress. In one case (#6), several oral temperatures readings were obtained, the highest being 37.1 °C, ruling out a diagnosis of heat stroke; nevertheless, the high level of solar radiation and the heavy workload justify the conclusion that heat stress was a contributing factor to death. In one case (#11), while heat stress was a possible contributory cause of death, there was evidence of snakebite as a more likely explanation.

In the 12 cases where a causal relationship with heat stress was certain or probable, Heat Index (which reflects shade temperature and humidity) ranged from 33 °C to 43 °C. (BOM data were not available in Case #10: this was in the engine room of a ship, where the reported air temperature was 48 °C). In the deaths that occurred following outdoor work, there was a significant additional heat load from solar radiation: the daily aggregate solar exposure ranged from 6.9 to 8.7 kWh·m^−2^. (As a guide to intensity of solar radiation, the mean solar load in the city of Adelaide, South Australia is 7.6 kWh·m^−2^ in January and 2.1 kWh·m^−2^ in June: this difference is of course partly due to the large seasonal variation in the number of daylight hours.)

### 3.3. Deaths in Which Heat Stress Was a Sufficient Cause

In two cases, drivers had become bogged in remote areas and set out on foot: modelling clearly shows that both would have incurred prolonged exposure to extreme heat with severe dehydration. While in one of these cases a severely dehydrated fellow worker was found in time to be resuscitated, these circumstances are incompatible with survival in the absence of rescue.

### 3.4. Deaths in Which Heat Stress Was not a Sufficient Cause

In the remaining 10 cases (that is, excluding the one case not attributable to heat stress), it is probable that heat stress was not the sole explanation. In six of these cases, this judgement is strengthened by the observation that there were fellow workers who were unaffected. Known risk factors for heat-related illness were identified in all cases, including reduced capacity for self-pacing, lack of acclimatisation, obesity, intercurrent illness and cardiovascular disease. Nevertheless, a complete explanation of the cause of death is lacking for two cases (#3 and #6), and, in one case (#8), there was significant trauma, suggesting that the environmental heat load was not the major factor in the death.

Four of the deaths followed indoor work.

### 3.5. Risk Factors

Inability to self-pace is a variable that is not measurable a priori: nevertheless, there were circumstances in two cases where there was a factor which self-evidently gives an incentive to override this safety mechanism: in one case, the subject was undergoing military duties in simulated battle conditions, and in another a door-to-door collector for charity was paid partly according to his success.

In six deaths the worker was in the first week of the job, indicating lack of acclimatisation as a risk factor.

One subject was reported as being obese. Two subjects were receiving anti-psychotic medication. In one subject, there was strong evidence of an intercurrent illness, possible myocarditis, and, in another, previously unrecognised pulmonary hypertension was a likely causal factor. Dilutional hyponatremia was confirmed in one case by low serum sodium (and low potassium); the worker had reported drinking large volumes of fluid.

### 3.6. Autopsy Findings

Autopsy details were available for seven of the cases. Cerebral oedema was found in two cases, and in a further two the brain weight was above normal values (1520 g and 1465 g) [7]. Pulmonary oedema was noted in six of these seven cases.

## 4. Discussion

The finding that heat stress was not the sole cause of 10 of the deaths indicates the importance of identifying risk factors in any workforce subjected to hot conditions.

### 4.1. Self-Pacing 

In at least two cases, self-pacing was compromised. The protective effect of self-pacing is most commonly overridden in sport, as evidenced by the often-reported occurrence of heat stroke in marathon runners [8]. In the occupational setting, attenuation of this protective mechanism can occur in military activity, where battle conditions or military discipline can inhibit the impulse to reduce physical workload. One such case occurred in this series, albeit in simulated battle conditions. Self-pacing may also be compromised under piecework conditions, as has been shown in studies of shearers and construction workers (although the consequences were not fatal) [3,4]. In this series, one death occurred in a collector for charity: his income was partly based on results.

### 4.2. Acclimatisation

A salient finding of this series was that in no fewer than six of the heat-related deaths the worker had been hired for less than a week. While this shows the importance of acclimatisation, it may also reflect compromised self-pacing: recently-hired workers may be especially motivated to push themselves beyond thermal comfort levels for fear of dismissal or of ridicule. This finding shows the need for special attention to recently-hired workers in hot weather. Expectations of work rates should be lowered during the acclimatisation period, and one-on-one supervision (the buddy system) is recommended.

### 4.3. Indoor Work

Four deaths occurred in the course of indoor work. Although indoor workers are spared the intense radiation load from solar exposure, they face the critical hazard of low air velocity: although the total heat load is less than with outdoor work, the capacity to dissipate body heat is reduced by the relatively still air, which reduces the evaporation of sweat. This is demonstrated in Appendix A by modelling, showing that the beneficial reduction in radiant heat load from working indoors can be offset by low air velocity, which reduces the capacity to evaporate sweat.

### 4.4. Intercurrent Illness

Case #2 appears to have had an intercurrent illness: some lymphocytes were present in the myocardium at autopsy, so that the worker’s condition may have been viral myocarditis. He had had almost no food or fluid intake over the four days leading to his death, and the likely dehydration, as well as the illness itself, were likely contributory factors. The lack of food intake over four days may also have reduced the worker’s heat tolerance. During work in the heat, there is normally an exaggerated rise in blood glucose, the source of which is breakdown of liver glycogen [9]. Liver glycogen levels run down quickly if carbohydrate intake is low or absent [10], thus it is quite likely that this worker’s tolerance to working in the heat was compromised by depletion of liver glycogen.

### 4.5. Obesity

Obesity, with accompanying lack of physical fitness, was a likely contributor to heat stroke in one case and is a well-recognised predisposing factor to heat-related disorders. In a series of fatal heat stroke cases, a 3.5-fold increase in risk was found in men 18 kg or more overweight compared with men who were 4.5 kg overweight [11]. The capacity to dissipate body heat by evaporation of sweat and convection is a function of skin surface area, whereas heat production is a function of body mass. Since obesity confers a lowered body surface area in relation to body mass, the capacity to dissipate heat is attenuated. Moreover, the extra weight carried by obese people requires more energy expenditure—hence more heat generation—to perform a given task compared with a lean individual [12,13].

### 4.6. Heart Disease

One subject was found at autopsy to have pulmonary hypertension with right ventricular hypertrophy. In another subject, discussed above, viral myocarditis was suspected. Cardiovascular disease has been observed to increase the risk of heat-related deaths. Cardiac output needs to increase in heat to increase perfusion of skin and increase sweat production. In heart disease, reduction in maximal oxygen uptake (VO_2_max) limits the capacity to increase cardiac output. During the European heat wave of 2003, daily mortality from cardiovascular disease in the German city of Essen was found to be 25% higher than the daily average over summer months [14].

### 4.7. Psychotropic Drugs

Two of the decedents were receiving psychotropic drugs, and in both instances the coroner gave much consideration to whether they had any causal role. Antipsychotic drugs have been associated with heat-related hospitalisation in a case-control study: the authors attributed the effect to the medication interfering with thermoregulation [15], although such dysregulation has also been attributed to mental disease itself [16]. On the other hand, convincing evidence of any effect of any individual psychotropic drug is lacking. One subject had been taking venlafaxine, clozapine and a low dose of amisulpride. The National Data of Adverse Event Notifications (DAEN) was consulted but this case was the only recorded occurrence of heat stroke or heat illness from clozapine: this subject had other risk factors. The other subject was taking quetiapine: although the coroner agreed with the forensic pathologist that this drug contributed to the worker’s death, interrogation of the DAEN identified only one notified case (non-fatal) of heat illness from quetiapine.

### 4.8. Hyponatremia

One subject, a soldier, was hyponatremic (#4): on admission to hospital, his serum sodium was 128 mm/L (reference range 135–145 mm/L). The serum potassium was also low (3 mm/L, reference range 3.5–5 mm/L), indicating that the low sodium level was dilutional. He had reported to the health centre in the middle of the day having drunk 8 L of fluid since 04:00. On admission to hospital that evening, his core temperature was 41.7 °C. Concurrent heat stroke and hyponatremia has previously been reported, also in a military situation [17].

Exertion-associated hyponatremia (EAH) is well recognised in marathon runners and in hikers [18] but is not common in the work environment. This is not surprising, since sweat has a lower sodium content than serum, so that uncompensated sweat loss leads to hypovolemic hypernatremia; only if the subject overcompensates by drinking excess water or hypotonic fluid can hyponatremia develop. It is commonly stated that the thirst mechanism is an inadequate driver for fluid replacement, and that drinking small amounts frequently is preferable to sole reliance on thirst [19]. On the contrary, an Exercise-associated Hyponatremia (EAH) Consensus Development Conference states “thirst should provide adequate stimulus for preventing excess dehydration and markedly reduce the risk of developing EAH in all sports” [20]. The occurrence in this series of a case of hyponatremia suggests the need for an awareness of risk from over-zealous fluid replacement.

### 4.9. Pulmonary and Cerebral Oedema

The findings of cerebral oedema and pulmonary oedema in heat stroke cases have been noted previously [21,22]. In a series of autopsies of military personnel who had died of heat stroke, the brain weight was usually higher than expected, often by several hundred grams, the average weight being 1493 g [23] compared with the average for normal adult males of 1336 g [7]. Since cerebral dysfunction is a constant feature of heat stroke, it is probable that cerebral oedema is the cause. In one case in this series, uncal and tonsillar herniation were found at autopsy, suggesting that the terminal event in some cases of heat stroke may be herniation into the foramen magnum from raised intracranial pressure. Consideration should therefore be given to specific remediation of cerebral oedema in hyperthermic individuals with signs of cerebral impairment.

## 5. Conclusions

The analysis supported the coroners’ conclusions that work-related heat stress was a causal factor in 12 of the 13 deaths. In only two cases heat stress was a sufficient cause of death, and risk factors other than heat stress were identified as potentially contributing to the other 10 deaths. The finding that 6 of the 13 deaths occurred in the worker’s first week on the job shows the need for special attention to recently-hired workers in hot weather. Expectations of work rates should be lowered during the acclimatisation period, and one-on-one supervision (the buddy system) is recommended. Other risk factors identified were compromised self-pacing, obesity, intercurrent illness and pre-existing heart disease. The autopsy finding of cerebral oedema is consistent with a large previous study. The possibility of uncal herniation as a precipitating cause of death suggests a possible benefit of measures to lower intracranial pressure in the emergency treatment of patients with heat stroke.

## Figures and Tables

**Table 1:** Summary of heat stress parameters and risk factors.

Study Number	Age	Activity	Max Heat Index	Daily Solar Exposure	Mean Air Velocity	Workload	Body Core Temperature	Time Employed	Heat Sufficient Cause?	Risk Factors	Fellow Workers
1	35	Stranded in outback, walking	43	8.1	7	Moderate	NR	1 day	Y	Dehydration	None present
2	30	Construction	36	6.3	22	Heavy	NR	3 days	N	Intercurrent illness, anorexia ?myocarditis	Unaffected
3	56	Door-to-door collection	36	7.0	38	Light -moderate	NR	Weeks or months	N	Piecework payment	None present
4	25	Military	37	6.9	NA	Moderate-heavy	41.7	Years	N	Hyponatremia	Unaffected
5	23	Supermarket trolley-boy	34	8.2	25	Moderate	40.5	4 days	N	Obesity, anti-psychotic medication	None present
6	38	Concrete formwork	41	8.7	17	Very heavy	37.1 (oral)	Weeks or months	N	Possible steroid use	Unaffected
7	25	Stranded in outback, walking	41	7.6	37.1	Moderate	NR	1 year	Y	Dehydration	Affected
8	50	Farm work	33	7.5	9	Low	NR	6 years	N	Prolonged exposure; trauma	None present
9	19	Installing insulation	39	Indoors	Indoors	Very heavy	40.5	1 day	N	Possible steroid use	Unaffected
10	25	Ship’s engine room	*	Indoors	Indoors	Moderate-heavy	41.5	4 days	N		Unaffected
11	34	Furniture removal	38	Indoors	Indoors	Moderate	42.3	1 day	N	Antipsychotic medication	Unaffected
12	24	Fruit- picking	33	8.2	15	Moderate	NR	2 days	N	Obesity, high humidity, suspected snakebite	Unaffected
13	72	Carpet-laying	36	Indoors	Indoors	Moderate-heavy		Not known	N	Pulmonary hypertension	None present

Units: heat index and body temperature in °C, daily solar exposure in kWh·m^−2^, air velocity in km/h. * No BOM data available: reported air temperature 48 °C. NR: not recorded.

## Appendix A

The effect of indoor work from the low air velocity is shown by modelling, in this case from the weather observations in Case #11.

The weather observations shortly before the worker became unwell were:

- Shade temperature (Ta) 37.4 °C
- Dewpoint 9.6 °C
- Wind speed 20.5 km/h
- Daily solar heat load 6.4 kWh·m^−2^

Since the solar load is high, mean radiant temperature (MRT) is approximately 75 °C.

Dew point of 9.6 °C equates to partial pressure of water vapour (PPA) of 9 mm Hg.

Using the experimentally-derived equations of Hertig [5],

Radiant heat load (R) = (MRT − Tsk) × 11.3 × k

where Tsk is skin temperature, i.e., 35 °C, and k is a function of clothing, assumed to be 0.7.

Then, when working outdoors

R = 11.3 (75 − 35) × 0.7 = 316 Kcal/h = 368 W,

for moderate workload. Metabolic heat load (M) = 300 W,

Maximum sweat evaporative capacity (Emax) = 2 × k × V^0.6^ × (42 − PPA)

where V = air velocity in m/min.

Thus, Emax = 2 × 0.7 × 20.5 × 1000/60 × (42 − 9) = 1530 Kcal/h = 1778 W.

Then, heat load as a proportion of capacity to dissipate heat (ignoring minor influence of convection) is

(R + M)/Emax = (368 + 300)/1778 = 37%,

whereas working indoors

Air velocity is typically no more than 1 m/s.

MRT is the same as Ta.

Thus,

R = 11.3 (37.4 − 35) × 0.7 = 16 Kcal/h = 18 W.

Emax = 2 × 0.7 × 60^0.6^ × (42 − 9) = 539 Kcal/h = 626 W.

Then,

(R + M)/Emax = (18 + 300) /626 = 318/626 = 51%.

This modelling, albeit simplified, shows that the beneficial reduction in radiant heat load from working indoors can be offset by the large reduction in air velocity.

## Appendix B

Case #1: A truck driver became bogged in a remote location. He abandoned his vehicle at about 15:00 and set out on foot. His body was found the following day, 30 km from the vehicle. It is not known whether the worker waited until evening before setting out on foot, but it is highly likely that he would have set out immediately so that he could follow the road. The dry-bulb temperature at 15:00 on the day when the worker was last seen alive was 42.2 °C, and on the following day the maximum dry-bulb temperature was 44.5 °C. Daily solar exposure was 8.1 kWh·m^−2^ on the first day and 7.5 kWh·m^−2^ on the second day. If he started walking straight away, his estimated hourly radiant heat load would be 413 W, in addition to the metabolic heat load of 300 W. Thus, the total evaporative sweat loss would have been well in excess of 1 L/h. Given the limited water supply he set out with, dehydration would soon have developed. The dehydration would have diminished his capacity for sweat production, with the probable consequence of heat storage. Death was almost certainly the result of the combined effects of dehydration and heat stroke. No pre-existing risk factors were identified, and it is therefore probable that in this case the heat stress was a sufficient cause of death: that is, nobody would be expected to survive in such circumstances. Had the worker stayed in the vehicle, with the windows open, his hourly total radiant and metabolic load would have been of the order of 200 W, with a fluid requirement from sweat evaporation of 300 mL/h, lessening with nightfall. With the air-conditioning functioning, fluid requirement would have been negligible. Thus, he would almost certainly have survived had he stayed with the vehicle.

Case #2: A bricklayer died after three days of work in moderately high heat (maximum dry bulb temperature 32.7 °C, solar exposure 470 W), high humidity (maximum dew point 22.5 °C) and mean air velocity 16.3 km/h. He ate almost no food in the four days prior to his death, so that there was a probable intercurrent illness. At autopsy, some lymphocytes were present amongst the myocardial fibres, and viral myocarditis was suspected. The level of heat and humidity suggest that the worker would probably have survived in cooler conditions. The lack of food intake would likely have led to some dehydration, as well as glycogen depletion: these would have been probable contributing factors, as well as the underlying illness, especially if it was myocarditis. Moreover, the deceased’s co-workers were not affected, thus it is highly likely that the intercurrent illness, as well as the lack of oral intake over four days, contributed to the death.

Case #3: A 56-year-old male died collecting donations door-to-door for a charity. He had been working for four days of a heat wave, daily maximum temperatures being 38.0, 41.0, 42.2 and 39.0 °C. Maximum dew point on the day he died was 19.6 °C, i.e., humidity was in the “uncomfortable” range, and daily solar exposure 7.0 kWh·m^−2^. The coroner’s report indicated that the worker was observed to be walking unsteadily on his feet shortly before he collapsed and died. He was mildly dehydrated, as suggested by the fact that his bladder was almost empty at autopsy. He was paid an incentive bonus on top of base salary, so there was a clear incentive to override the protection of self-pacing. Autopsy showed minimal atherosclerosis of the coronary vessels, and the prior observation of walking unsteadily makes sudden onset of ventricular fibrillation unlikely. Thus, while the oppressive heat conditions and the risk factors of dehydration and compromised self-pacing implicate heat stress as a causal factor, a complete explanation for this death is lacking, given the workload which was moderate only.

Case #4: A 25-year-old soldier died on a training course. For the previous two weeks, he had been engaged in outdoor activity. Temperature and humidity were high. There was no local BOM monitoring station but maximum WBGT measured on-site was 36 °C at 11:40. Work had commenced at 05:00 and continued until a scheduled rest period from 10:00 to 15:00. At 12:25, he had presented to a medic, having vomited and feeling breathless. He had drunk 8 L of fluid since 04:00. His temperature was 37.8 °C but settled to 37.1 °C, and at his request he was allowed to return to work, which he did at 15:30. He became disoriented, lost consciousness and was transferred to hospital with a core temperature of 41.7 °C on admission and died that evening. Serum electrolyte analysis was performed on a blood sample taken when he was admitted to hospital: serum sodium level was 128 mmol/L, which is below the normal range of 135–145 mmol/L, thus indicating hyponatremia. Although there is no indication of other soldiers being affected that day, another had suffered severe heat stroke five weeks before, and other course participants had suffered heat stroke in previous years. The motivation to return to duties implies compromised self-pacing, which is common in a military situation. Although these were only simulated battle conditions, completion of the course was a pre-requisite for promotion. Heat stroke is confirmed by the core temperature of 41.7 °C, so that the extreme conditions undoubtedly contributed to this death. As to why this soldier was affected and others were not, hyponatremia is a probable cause, suggested by the large fluid intake in the morning and his reported breathlessness, and confirmed by the low serum sodium concentration and marked pulmonary congestion noted at autopsy. Although cerebral oedema was not reported at autopsy, the brain weight of 1520 g is above expected value.

Case #5: A 23-year-old male collapsed working as a supermarket trolley boy. It was his fourth day at the job, following chronic unemployment due to a psychiatric disorder for which has was taking clozapine, amisulpride and venlafaxine. There was concern about his ability to perform the work because of his obesity. Maximum air temperature was 30.9 °C, humidity was high (maximum dew point 23.9 °C), moderate air velocity (21 km/h), and high radiant heat exposure (daily solar exposure 8.2 kWh·m^−2^) He did not eat lunch and worked through the lunch break. He was noted to have a high temperature, but the reading is not recorded. In hospital, the worker developed multiple organ failure and died 12 days later. Likely factors in the death were high radiant heat load, high humidity, compromised self-pacing (probably having been conscious of his job security during this closely monitored trial of work), obesity and lack of fitness. The role of the antipsychotic medication is uncertain.

Case #6. A 38-year-old man died after working on concrete formwork. He was probably acclimatised but this was his first day back after a weekend break. Maximum dB was 41.4 °C, maximum dew point 19.3 °C, and daily solar exposure 8.7 kWh·m^−2^. He was very muscular and appeared to have been taking “supplements”, but it is not stated what they were, and blood toxicology was not informative. He had become unwell and went to the health centre where a high breathing rate was noted, but temperature was normal (36.1 °C oral). He skipped the evening meal, and at 20:00 became incoherent and unsteady. Oral temperature again was normal. He was transported to hospital but died *en route*. At autopsy, there was swelling of the internal organs and flattening of the sulci of the brain. Sodium and potassium levels were measured in the vitreous humour and were elevated. Other workers also had found the heat oppressive, but there is no indication that any became unwell. Although normal oral temperature makes a diagnosis of heat stroke unlikely, it is probable that the heavy radiant heat exposure and the high work rate were causal factors in his death. (The inquest in this case has been re-opened.)

Case #7. A 25-year-old male, with a co-worker as passenger, was driving a vehicle which became bogged in a remote location. He and the co-worker left the vehicle, carrying 1.5 L of water. He was found deceased some hours later, 6.7 km from the vehicle. The co-worker was found severely dehydrated soon after: he was rehydrated and survived. The coronial report states that air temperature was 47 °C. The nearest BOM station, 100 km away, recorded a maximum temperature of 43.2 °C. Humidity was low (dew point 12.6 °C), and daily solar exposure 7.6 kWh·m^−2^. It is thus likely that the mean radiant temperature was close to 80 °C, which would lead to an hourly radiant heat load of 413 W for a person in the open. With an additional metabolic heat load of 300 W from walking, the total heat load of 713 W would require evaporation of more than 1 L/h (disregarding convective heat load). Had he remained in the vehicle with the windows open or had he found some shade to sit in while waiting to be rescued, the hourly heat load would have been 225 W with a fluid requirement of 350 mL; and if he had remained in the vehicle with the air-conditioning on, his fluid requirement would be close to zero. In either case, he would most probably have survived until rescued.

Case #8. A 50-year-old farmer was impaled by farm machinery—caught in his foot—on a warm February day. He was found deceased at 21:30 the following night, i.e., about 30 h from when he was last seen. The coroner found that death was caused by shock and exposure. Maximum air temperature was 34.6 °C on the first day, and daily solar exposure was 7.5 kWh·m^−2^. On the second day, maximum air temperature was 35.5 °C, and daily solar exposure 7.3 kWh·m^−2^. The coroner concluded that death probably occurred on the first day. Although heat exposure and the consequent dehydration probably contributed to this death, the primary causes were the impaling injury causing shock and immobility, and that he was not rescued earlier. It is likely that he would have survived longer if the environmental heat levels had been lower, but, even if the accident had occurred on a cool February day, it is not possible to say whether he would have survived for the 30 h between the injury and the arrival of help.

Case #9: A 19-year old male died after performing very heavy work installing insulation in a roof space for about 1.5 h. It was his first day in the job. Maximum dB was 40.5 °C, DP 18.7 °C, but mean hourly solar exposure was only 4.7 kWh·m^−2^, possibly because of cloud cover. Air speed was low (mean 11 km/h) and work was performed mainly under shade (in the roof space installing insulation). There was pressure to complete the job expeditiously. The one co-worker was unaffected. Heat stroke was confirmed by body core temperature of 40.5 °C and the onset of total organ failure, rhabdomyolysis and coagulopathy. The prime factors were the low air velocity associated with indoor work, which severely limits sweat evaporative capacity. Other factors were high ambient temperature, compromised self-pacing and lack of acclimatisation.

Case #10. A 25-year-old cadet engineer and three other marine personnel were replacing a valve *n* the engine room of a ship, located in waters close to Vietnam. Work commenced at 08:30. The ambient temperature in that location was said to range 35–50 °C. No meteorological data were available, but the engine room temperature was reported to be 48 °C with high humidity. An air-conditioned retreat area was close by. The cadet engineer had joined the vessel four days previously. At 10:30, he became disoriented and complained of feeling hot. Sweating was absent. A body temperature of 41.5 °C was recorded, with heart rate 166/min, blood pressure 126/69, and shallow laboured respiration. He died at 12:05. Autopsy showed gross cerebral oedema with uncal and tonsillar herniation, and gross oedema of the lungs. There was no mention of other workers being affected. It was thought that the deceased worker had not been taking rest breaks or drinking enough water.

Case #11. A 34-year old male became unwell at the end of a day in furniture removal. It was the third day of a heat wave, but he was only employed occasionally so that this appears to have been his first day working in the heat. Air temperature was 40.1 °C at 15:00, 37.4 °C at 18:00 and 29.3 °C at 21:00. The worker became ill at about 19:00 and was taken to hospital. His highest recorded body temperature was 42.3 °C. He died six hours after admission to hospital. Although he had been assigned relatively light furniture items to carry, it is likely that his workload was moderately heavy. Since a large amount of the work was performed indoors, the radiant heat load would have been much less than for outdoor work. In this case, a critical factor is the greatly reduced air velocity indoors, which reduces the convective heat loss and the capacity to evaporate sweat. The high body temperature confirms the diagnosis of heat stroke, and the high air temperature and the moderately heavy workload being performed largely indoors would have been significant causal factors. The fact that there were fellow workers unaffected suggests that there were personal factors making this worker more vulnerable to heat strain. Self-pacing was compromised: the worker had kept working despite some signs of unwellness earlier in the day. Lack of acclimatisation was another likely factor, this being his first day on this job. The worker was on regular antipsychotic medication, and lack of physical fitness is common in psychiatric illness. The medication was diazepam and quetiapine, and the coroner believed that the quetiapine was a causal factor in this worker’s death. There is some epidemiological evidence that antipsychotic medication can predispose to heat strain (discussed above), although there is limited evidence specifically related to quetiapine. The national Database of Adverse Event Notifications (DAEN) has only one record of heat stroke in a person taking quetiapine (it is probably not this worker as it was not a fatal event). Two witnesses mentioned that the worker had drunk a large amount of water, so that dehydration was probably not a factor. Indeed, his serum sodium level was 132 mm/L on admission to hospital (normal range 135–145), but the potassium level was elevated: thus, the hyponatremia was likely not dilutional but a consequence of the general metabolic failure from the hyperthermia (there was also biochemical evidence of renal failure and incipient hepatic failure).

Case #12. A 24-year-old woman visiting Australia on a working visa began work at 10:00 cutting wires on a tomato plantation. She had commenced work on the previous day at a watermelon farm. At 12:30 she complained of feeling hot and thirsty. She became disoriented then lost consciousness and breathing ceased. She did not respond to cardiopulmonary resuscitation (CPR). Body temperature was not recorded. Air temperature was 31.3 °C at 12:00, with moderate humidity (dew point 18.5 °C with heat index 34 °C. Solar exposure was very high—8.2 kWh·m^−2^. At autopsy obesity was noted. The lungs were heavy with haemorrhagic oedema. There were two small red puncture marks on the right lower leg, about 0.8 cm apart, suggestive of snakebite.

Case #13. A 72-year-old man was contracted to replace a carpet in one room of a rental property. He commenced working, alone, at 12:30, and was found deceased at 16:03 with a hammer in his hand, suggesting sudden death. Air temperature was 38.1 °C at 12:00 and 35.2 °C at 15:00. Humidity was low (dew point 0 °C and 5 °C respectively), and total daily solar exposure was 8.9 kWh·m^−2^. There were mild emphysematous changes in the lung. There was evidence of pulmonary hypertension, with right ventricular dilatation and hypertrophy of the right ventricle and septum. Since this was indoor work, the likely low air velocity would have limited his sweat evaporative capacity. The heart condition found at autopsy would have limited his VO_2_max.
